# Supplementary material for: Complexome profiling of the Chlamydomonas psb28 mutant reveals TEF5 as an early PSII assembly factor
Source: Plant Cell. 2025 Mar 17;37(6):koaf055. doi: 10.1093/plcell/koaf055 (PMC12164590; doi:10.1093/plcell/koaf055)
Supplement: koaf055_Supplementary_Data [file koaf055_supplementary_data.zip › psb28_interactive.zip!/plots/intensity/qualityPlots/peptides/_Cre06.g292400.t1.1.html]

### Cre06.g292400.t1.1

Trivial name:   
SeqCoverage [median]: 22.90  
Mass: 34.50 kDa  
  
 

| Sequence | MissedCleavage | Mass [Da] | wtIntensity | muIntensity | Score |
| --- | --- | --- | --- | --- | --- |
| ADYDEQYFVR | 0 | 1304.57 | 413.40 | 6945.61 | 125.82 |
| AEILASIR | 0 | 871.51 | 255.21 | 0.00 | 71.50 |
| AQRGDGPSTSGR | 1 | 1187.56 | 287.34 | 932.16 | 79.09 |
| GATAPDGATDPEVGAGLK | 0 | 1625.79 | 428.52 | 2145.95 | 92.92 |
| LAAAGGTTHVIDPAR | 0 | 1448.77 | 3952.88 | 8233.80 | 59.90 |
| VVAQLFTPAAK | 0 | 1143.67 | 322.11 | 8925.22 | 122.13 |
|  |  |  | 5659.4 | 27182.7 |  |
